# Obesity and Urban Environments

**DOI:** 10.3390/ijerph16030464

**Published:** 2019-02-05

**Authors:** Peter Congdon

**Affiliations:** School of Geography, Queen Mary University of London, Mile End Rd, E1 4NS London, UK; p.congdon@qmul.ac.uk

**Keywords:** obesity, urban configuration, sprawl, food desert, fast food, exercise access, social environment

## Abstract

Obesity is a major public health issue, affecting both developed and developing societies. Obesity increases the risk for heart disease, stroke, some cancers, and type II diabetes. While individual behaviours are important risk factors, impacts on obesity and overweight of the urban physical and social environment have figured large in the recent epidemiological literature, though evidence is incomplete and from a limited range of countries. Prominent among identified environmental influences are urban layout and sprawl, healthy food access, exercise access, and the neighbourhood social environment. This paper reviews the literature and highlights the special issue contributions within that literature.

## 1. Background

Obesity is a major public health issue, affecting both developed and developing societies [1]. Obesity increases the risk for heart disease, stroke, some types of cancer, and type II diabetes. While individual behaviours are important risk factors, impacts on obesity and overweight of the urban physical and social environment have figured large in the recent epidemiological literature, though evidence is incomplete and from a limited range of countries.

## 2. Trends in Obesity

Although genetic factors affect susceptibility to obesity [2], they cannot account for the rapid increases in obesity in recent decades. Instead, changes in physical activity and diet are the focus in explaining this adverse trend [3]. Physical activity levels are declining, and sedentary behaviours increasing, not only in developed countries, such as the U.S., but also in low- and middle-income countries, such as China [4,5]. Dietary changes, such as the growth in fast food consumption and the “nutrition transition” [6,7] are also significant, and again affect both developed and less developed societies.

In turn, environmental factors are important influences on changing levels of physical activity and on changing dietary behaviours throughout the life course [8]. Variations in the impact of these factors according to socioeconomic level, ethnicity, and geographic location are a major focus in obesity research [9,10].

Regarding environmental influences on physical activity, one may distinguish [11] between proximal (home) and distal (neighbourhood) environmental and social factors. Proximal factors include influences on sedentary behaviour during childhood, and activity levels across the life course [12,13,14,15]. Prominent among identified distal environmental influences are urban layout, healthy food access, exercise access, and the neighbourhood social environment.

## 3. Urban Layout and Physical Activity

Especially in the US, Canada, and Australia, a large literature concerns urban configuration in terms of its impact on physical activity, framed especially in terms of neighbourhood walkability, and levels of active commuting [16,17,18].

Thus, sprawl has been defined as low density suburban development, with segregated land uses, low connectivity [19], high automobile dependence, and disincentives to physical activity [20,21]. By contrast, walkable neighbourhoods facilitate walking or bicycling to workplaces and to amenities such as shopping centres, parks, schools, and entertainment centres, rather than requiring automobile trips. Thus, [22] in a time series analysis of Ontario neighbourhoods, report no increase in obesity and overweight in more walkable neighbourhoods, whereas increases occurred in less walkable neighbourhoods.

A study of active commuting in a low- to middle-income country is exemplified by Adlakha et al. [23] with a focus on Chennai, India, where rapid growth in car ownership has reduced active commuting. They report that active commuting and use of public transport is associated with a greater mix of land uses, but not with street connectivity.

## 4. Environment and Diet

Regarding environmental influences on dietary patterns, the local food environment and access to healthy food outlets is a major research focus. Thus, food deserts have been defined as areas with diminished access to fresh fruit, vegetables, and other whole foods, and tending to be found in socio-economically deprived areas or ethnic minority neighbourhoods [24,25,26].

This is taken to reflect the location of supermarkets, grocery stores, and farmers’ markets, as opposed to fast food outlets and convenience stores offering processed food with high sugar and fat content. In USA, the USDA estimates that 23.5 million people live in urban neighbourhoods and rural towns with limited access to fresh, affordable, healthy food. On the other hand, sceptical studies regarding the impact of the neighbourhood food environment have emphasized instead demand differences between income groups [27].

As well as neighbourhood access to healthy food, access of schools to healthy food options is also a considerable research focus—given that increases in child and adolescent obesity have been a feature of the overall growth in obesity, and may be linked to deprivation [28]. A Dutch study [29] found that, in general, unhealthy options (e.g., fried snacks, sugar-sweetened beverages) were more often in close proximity to schools in comparison with healthy options. Moreover, fast food outlets were more often in the vicinity of secondary schools in lower SES neighbourhoods (28.6%) than in higher SES neighbourhoods (11.5%).

Studies such as that by Murphy et al. [30] in Melbourne consider the interplay between sprawl and food access. This study reports worse access to supermarkets, and higher BMI, in low-density residential development in contrast to compact higher density areas.

## 5. Environmental Justice and Access to Physical Activity

In general, studies predominantly from Australia, the UK, and the USA have demonstrated that living in closer proximity to public open space (POS) is associated with greater physical activity and improved health, for all age groups. However, access to POS varies by social group in line with a broader theme of environmental injustice, namely disproportionate exposure to adverse environmental conditions experienced by low-income and ethnic minority groups.

For example, there is evidence that the distribution of urban green space and parks [31] disproportionately benefits higher income and white ethnic groups. Both access and quality of parks may depend on the socio-economic level of neighbourhoods [32]. Methods for measuring access itself are important: a Mediterranean study [33] interrelates walkability and POS access, using three objective indicators of exposure to POS: “the distance of the walkable street network to the closest POS; the number of POS and the total area of POS within each network walkable street buffers (0.5 km, 1 km and 1.5 km) of participants residential addresses, using only the walking and/or cycling street network, ignoring routes restricted to pedestrians”.

## 6. Neighbourhood Social Environment

While the neighbourhood built environment has been extensively studied in relation to obesity, the neighbourhood social environment is perhaps an equally important component of the neighbourhood environment that is relevant to obesity among both adults and children. This includes aspects such as social capital, collective efficacy, and crime [34]. Thus, Holtgrave and Crosby [35] report that multivariate linear regression showed social capital to be a predictor of both obesity and diabetes (explaining 10% of the variance in obesity and 44% of the variance in diabetes). A Scottish study [36] examined the effects on adiposity of cumulative exposures to adverse neighbourhood conditions (as reflected in adverse neighbourhood perceptions). They found stronger relationships for abdominal obesity and percentage body fat, and weaker relationships with BMI, in line with a mechanism in which prolonged stress activates the hypothalamic–pituitary–adrenal axis and in turn leads to increased abdominal obesity.

## 7. Analytic Approaches

Analytic approaches to studying environmental impacts may be best provided by multilevel frameworks accounting for contextual risk factors beyond the individual, including the home, parental characteristics (for child subjects), and the physical and social environment [37,38,39]. Thus, Yip et al. [40] found that after adjusting for age, sex, income, education, and urbanity, both individual-level and community-level social cohesion were positively associated with physical activity.

Studies with a primarily geographic focus are also relevant, for example, to assessing urban–rural contrasts [41] or the obesity epidemic in developing countries [42]. A study of US counties [43] considered aspects of urban settlement and commuting (namely car commuting), healthy food availability, and exercise access. These factors were able to explain over 50% of the variability in obesity, and accounted for much of the spatial clustering evident in the obesity maps. A Korean study [44] considered spatial variability in the impacts of area risk factors. It found environmental factors to have stronger effects on local obesity rates for women than for men (as also reported in [43]), and that environmental factors have more spatially varying effects on local obesity for women than for men.

## 8. Debated Questions

A range of issues are still open to research and debate. For example, there is much discussion over the relative importance of exercise and diet in the development of obesity. Some reviews [45,46] stress dietary changes, others stress the role of physical activity [47,48]. Similarly, the concept of food deserts, and of diminished food choices in lower income areas, has been questioned in some studies [27,49,50]. The appropriate focus in obesity interventions is also currently being discussed. For example, a Spanish study [51] emphasizes food access and improved access to POS and sports facilities, while other studies [52] put focus on health-promoting urban design and improved walkability.
